# Resolving intergenotypic *Striga* resistance in sorghum

**DOI:** 10.1093/jxb/erad210

**Published:** 2023-06-01

**Authors:** Sylvia Mutinda, Fredrick M Mobegi, Brett Hale, Olivier Dayou, Elijah Ateka, Asela Wijeratne, Susann Wicke, Emily S Bellis, Steven Runo

**Affiliations:** Pan African University Institute for Basic Sciences, Technology and Innovation, Nairobi, Kenya; Department of Biochemistry, Microbiology and Biotechnology, Kenyatta University, Nairobi, Kenya; Department of Clinical Immunology, PathWest Laboratory Medicine WA, Fiona Stanley Hospital Network, Murdoch, Western Australia; Department of Biological Sciences, Arkansas State University, Jonesboro, AR, USA; Institute for Biology, Humboldt University, Germany; Department of Horticulture, Jomo Kenyatta University of Agriculture and Technology, Nairobi, Kenya; Department of Biological Sciences, Arkansas State University, Jonesboro, AR, USA; Institute for Biology, Humboldt University, Germany; Department of Computer Science, Arkansas State University, Jonesboro, AR, USA; Department of Biochemistry, Microbiology and Biotechnology, Kenyatta University, Nairobi, Kenya; Institute for Biology, Humboldt University, Germany; University of Ghent, Belgium

**Keywords:** Cell wall-based resistance, comparative transcriptomics, lignin-based resistance, parasitic plants, pathogen-associated molecular patterns, programmed cell death, weighted gene co-expression networks

## Abstract

Genetic underpinnings of host–pathogen interactions in the parasitic plant *Striga hermonthica*, a root parasitic plant that ravages cereals in sub-Saharan Africa, are unclear. We performed a comparative transcriptome study on five genotypes of sorghum exhibiting diverse resistance responses to *S. hermonthica* using weighted gene co-expression network analysis (WGCNA). We found that *S. hermonthica* elicits both basal and effector-triggered immunity—like a bona fide pathogen. The resistance response was genotype specific. Some resistance responses followed the salicylic acid-dependent signaling pathway for systemic acquired resistance characterized by cell wall reinforcements, lignification, and callose deposition, while in others the WRKY-dependent signaling pathway was activated, leading to a hypersensitive response. In some genotypes, both modes of resistance were activated, while in others either mode dominated the resistance response. Cell wall-based resistance was common to all sorghum genotypes but strongest in IS2814, while a hypersensitive response was specific to N13, IS9830, and IS41724. WGCNA further allowed for pinpointing of *S. hermonthica* resistance causative genes in sorghum, including glucan synthase-like 10 gene, a pathogenesis-related thaumatin-like family gene, and a phosphoinositide phosphatase gene. Such candidate genes will form a good basis for subsequent functional validation and possibly future resistance breeding.

## Teaser

Parasitic plants of the *Striga*genus are major pests to cereals in Africa. We pinpointed genetic causes of *Striga*resistance in sorghum that can be harnessed for future resistance breeding.

## Introduction

Sorghum [*Sorghum bicolor* (L) Moench] is a staple cereal for millions in sub-Saharan Africa (SSA) but, its production is greatly constrained by the parasitic plant *Striga* spp. (*Orobanchaceae* family). *Striga* attaches to roots of host crops to siphon water and nutrients. In sorghum, *Striga* leads to crop losses of between 30% and 100% (Ejeta, 2007), exposing millions of families that depend on the cereal to hunger and loss of livelihoods.

*Striga* is difficult to control because of its well-adapted lifestyle (Runo and Kuria, 2018). Parasitism is tightly cued to the host, allowing seed germination only in response to host-derived biomolecules, hormones—mainly strigolactones (Matusova *et al*., 2005; Bouwmeester *et al*., 2007; Gomez-Roldan *et al*., 2008; López-Ráez *et al*., 2011). Germination is followed by the parasite attaching to the host and developing vascular vessels that create a continuum for water and nutrient acquisition (Dörr, 1997). Parasitism continues for 30–40 d below-ground, after which *Striga* emerges from the soil and becomes photosynthetic. *Striga* then flowers to complete its life cycle by producing ~50 000 seeds per flower (Berner *et al*., 1995).

Smallholder farmers in SSA try to maintain low *Striga* infestation levels using agronomic and cultural practices such as hand weeding and crop rotation with non-hosts (Kanampiu *et al*., 2018), but these have achieved little or no success. A more practical and sustainable strategy would be to integrate multiple management approaches and greatly leverage natural host resistance (Mwangangi *et al*., 2021).

However, mechanics of host resistance against *Striga* are only beginning to be unraveled. At the fore are new findings that show *Striga* spp. to be sophisticated manipulators of plant cells (Li *et al*., 2009), and the parasite’s ability to trigger the host innate immune response (Su *et al*., 2020) reminiscent of host–pathogen interactions (Dangl and Jones, 2006). If this model fits *Striga*–host interaction, *Striga* should firstly elicit pathogen-triggered immunity (PTI) through its as yet to be determined pathogen-associated molecular patterns (PAMPs) that make it encounter mechanical barriers from the host, manifesting in the form of cell wall and biochemical fortifications. *Striga* should then suppress PTI and promote pathogenesis by a second offensive on the host through injection of effector-like molecules into plant cells (Dangl and Jones, 2006). If the host is resistant, effector-triggered immunity (ETI)—whose hallmark is programmed cell death—will be activated, effectively stopping further parasitism.

Responses suggestive of PTI and ETI have been reported in various sorghum genotypes; for instance mechanical barrier-type resistance that fortifies the host cell wall to block ingression of the parasite in the east African Durra sorghum N13 (Maiti *et al*., 1984; Mbuvi *et al*., 2017; Kavuluko *et al*., 2021), programmed cell death at the interface of the host and the parasite that is characterized by a HR at the host–parasite junction in IS14963 (Kavuluko *et al*., 2021), and deposition of secondary metabolites such as polyphenols at the interface of the host and the parasite, in some wild sorghum accessions (Mbuvi *et al*., 2017).

What is not clear is whether specific sorghum genotypes exhibit one or more modes of resistance working in concert to ward off *Striga*. So far, data favor a model in which a genotype has one—or a dominant—mechanism of resistance. Genetically, such a resistance model is suggestive of multiple independent pathways activating *Striga* resistance—harbored in different genotypes. Here, we use a comparative transcriptome analysis of sorghum harboring distinct phenotypes to clarify if multiple forms of resistance can manifest in the same genotype against the alternative, that genotypes have predominantly one mode of resistance. Our findings have important implications in breeding for broad-spectrum and durable *Striga* resistance in sorghum.

## Materials and methods

### Plant materials

We selected sorghum genotypes representing mechanical barrier resistance—a HR—then used a comparative transcriptomics approach to hone in on the genetic underpinnings of each resistance mechanism. Genotypes comprised the east African Durra sorghum N13, Middle East Durra-Caudatum, IS2814, and the advanced east African Caudatum IS9830 to represent mechanical barrier resistance (Maiti *et al*., 1984; Mbuvi *et al*., 2017; Kavuluko *et al*., 2021), and IS14963, a Caudatum from Central Africa, and IS41724, an Indian Durra, to represent hypersensitive interactions. These sorghum materials were originally obtained from the International Crops Research Institute for Arid and Semi-arid Tropics (ICRISAT) and are maintained at the Plant Transformation Laboratory (PTL) at Kenyatta University. Seeds of *S. hermonthica* were obtained from infested farms in western Kenya Busia, Alupe (0.45°, 34.13°) in 2018. Only *S. hermonthica* seeds were used in this study.

### Conditioning and germination of *Striga* seeds

Conditioning of *Striga* seeds was done according to Mbuvi *et al*. (2017). Prior to conditioning, *Striga* seeds (25 mg) were washed in 25 ml of 10% (v/v) sodium hypochlorite (commercial bleach) for 10 min with gentle agitation. Seeds were collected in a funnel lined with Whatman GFA filter paper (Meadow, UK) and thoroughly rinsed with double-distilled water to remove the sodium hypochlorite. Seeds were then transferred on Petri plates (90 mm) lined with moisturized filter papers and incubated in the dark at 30 °C for 14 d. After conditioning, the seeds were pre-germinated by adding 3 ml of 0.1 ppm GR24 (Chiralix, Nijmegen, the Netherlands) and incubating overnight at 30 °C.

### Sorghum germination and infection with *Striga*

Sorghum seeds were sown in germination pots (10 × 10 × 7 cm) filled with moisturized vermiculite and watered with Long Ashton nutrient media (Hudson, 1967). Ten days following germination, seedlings were transferred to rhizotrons which are soil-free transparent root analysis chambers of the dimensions 25 × 25 × 5 cm (Nunc, ThermoFisher Scientific, UK). The chambers were prepared as described by Mbuvi *et al*. (2017) by filling them with vermiculite and then overlaying them with 50 μm thick nylon mesh. The chambers were then wrapped with aluminum foil and kept in a glasshouse at temperature cycles of 28 °C day:24 °C night with a 12 h photoperiod and 60% humidity. During this period, the plants were drip-fed with nutrient media. To infect sorghum roots with *Striga*, rhizotrons were opened and sorghum roots carefully aligned with pre-germinated *Striga* seeds using a soft paint brush. After infection, the chambers were closed, wrapped in aluminum foil, and maintained in the glasshouse as described above. Three plants per genotype were screened in a randomized complete block design. Plants were kept in a glasshouse at temperature cycles of 28 °C, day and 24 °C night with 12 h photoperiods.

### Characterizing *Striga* resistance mechanisms in sorghum

Mechanisms of resistance against *Striga* were evaluated in two genotypes, namely IS2814 and IS41724, using histological assays. These genotypes had previously been reported to harbor post-attachment resistance based on low *Striga* number, short length, and low biomass metrics (Kavuluko *et al*., 2021). Histological analyses were done by studying the host–parasite interface, 9 days after infection (dai) as described by Kavuluko *et al*. (2021). Briefly, tissue segments at the sorghum–*Striga* interface were dissected, fixed in Carnoy’s fixative, and stained with 1% safranin O dye. Tissues were then cleared of safranin O dye in chloral hydrate solution (2.5 g ml^–1^) overnight with gentle shaking. Following de-staining, photographs revealing the extent of parasite ingression on the host roots were documented using a Leica stereomicroscope MZ10F fitted with DFC 310FX camera.

Prior to sectioning, fixed tissues were pre-processed using the Technovit 7100™ kit following the manufacturer’s instructions (Heraeus Kulzer GmbH, Hanau, Germany) and protocols described in Mbuvi *et al*. (2017). The tissues were pre-infiltrated (1:1, Technovit™ solution:100% ethanol), infiltrated (100% Technovit solution), embedded in 1.5 ml microcentrifuge tube lids containing Technovit®1/Hardener 2 (1:15), and left to set for 2 weeks. Embedded tissues were mounted onto wooden blocks using the Technovit™ 3040 kit following the manufacturer’s instructions (HaraeusKulzer GmbH). The wooden blocks holding the tissues were set on the rotary Leica RM2145 microtome (Leica, Germany) and 5 μm thick sections were obtained. Sections were placed on hydrated glass slides, dried on a hot plate at 65 °C, stained using 0.1% toluidine blue O dye in 100 mM phosphate buffer for 2 min, then washed in distilled water. A drop of DePex (BDH, Poole, UK) was applied on each of the dry sections and overlaid with coverslips. The slides were observed and photographed using a Leica DM100 microscope fitted with a Leica MC190 HD camera (both Leica, Germany).

### Lignin staining

Surface-sterilized sorghum seeds of the genotypes N13, IS41724, and IS14963 were germinated and transferred to rhizotrons. Sorghum roots were allowed to spread followed by infection with pre-germinated *Striga* seeds. At 9 dai, root segments were excised at the point of *Striga* attachment, embedded in 7% (w/v) agar, and sectioned by a vibratome (Leica VTI200s, Leica Germany). Sections 20 μm thick were stained with 1% (w/v) phloroglucinol solution for 10 min prepared as follows: 0.3 g of phloroglucinol was dissolved in 10 ml of absolute ethanol to make a 3% phloroglucinol solution which was then mixed with 2 M HCl in a ratio of 2:1 to make a 1% staining solution. Photographs were taken using the Leica DM100 microscope fitted with a Leica MC190 HD camera (Leica, Germany).

### RNA isolation, library preparation, and sequencing

Root tissues were collected from sorghum infected with *Striga* at 3 and 9 dai. Non-infected tissues corresponding to 9 dai were also collected from each genotype. Three replicates were collected for each treatment in the five genotypes. Samples were immediately frozen and ground in liquid nitrogen. RNA isolation was carried out using the ISOLATE II RNA Plant kit (Bioline, Meridian, UK) according to the manufacturer’s recommendations. Samples were extracted in Trizol including on-column desalting, DNase treatment, and purification. The quantity and quality of the isolated RNA were assessed by nanodrop and Agilent Bioanalyzer [RNA integrity number (RIN)], respectively. Samples with a RIN value of ≥7.0 were used to prepare Tag Seq libraries according to Lohman *et al*. (2016).

### Quality control pre-processing of raw 3ʹ Tag-RNA-Seq reads

The 3-Tag-RNA-Seq-analysis Next Flow pipeline (https://github.com/fmobegi/3-Tag-RNA-Seq-analysis) which wraps up the TagSeq utilities v2.0 (https://github.com/Eli-Meyer/TagSeq_utilities) was used to process the RNA-seq data. Briefly, the quality of raw sequences was assessed using ‘FastQC’ v.0.11.9. Raw reads were filtered based on Phred per-base quality score (Q) ≥20 and a cumulative per-read low quality score (LQ) ≤10 using the QualFilterFastq.pl script with the parameters -m 20 and -x 10. Reads that passed this step were then depleted of homopolymer repeats longer than 30 bp using HRFilterFastq.pl (parameters -n=30), adapter sequences using BBduk.sh, part of BBMap v.35.85, and PCR duplicates using RemovePCRDups.pl. Non-template sequences introduced at the 5ʹ end of cDNA tags during Tag-Seq library preparation were trimmed using TagTrimmer.pl (parameters: -b 1 -e 8). Quality-processed reads were re-evaluated for quality using FastQC (https://www.bioinformatics.babraham.ac.uk/projects/fastqc/) and then mapped using HISAT (Kim *et al*., 2015) to the *Sorghum bicolor* reference. SAMtools v.1.10 (Etherington *et al*., 2014) was used to convert the generated sequence alignment map (SAM) files into binary alignment mapping (BAM), and to sort and index the BAM files. Transcripts were quantified using StringTie v.2.1.1 (Pertea *et al*., 2014) to generate normalized counts in the form of fragments per kilobase of transcript per million mapped reads (FPKM) and transcripts per million (TPM) in addition to RAW counts for differential gene expression analysis.

### Identification of differentially expressed genes

The workflow for analysis of differentially expressed genes (DEGs) is provided in our GitHub (https://github.com/fmobegi/3-Tag-RNA-Seq-analysis). Raw counts were transformed into counts per million (CPM) using the cpm() function in the edgeR package (Robinson *et al*., 2010). Genes with a CPM >1 were retained for further analysis. Principle coordinate analysis (PCoA) plots were generated using ggplot2 (Wickham, 2011) to determine the relatedness of the biological replicates.

Differential gene expression analysis was performed using the DESeq2 Bioconductor R package following normalization using DESeq2 (Love *et al*., 2014) with a Wald test. A false discovery rate (FDR) cut-off of 0.05 was applied, and a log2 fold change (FC) cut-off of ≥2 to indicate up-regulation and ≤ −2 to indicate down-regulation. DEGs were considered at each time point for each host in relation to the controls and displayed as heatmaps using the ‘pheatmap()’ function in R. UpSet plots were then used to visualize in the numbers of DEGs.

### Weighted gene co-expression network analysis

Weighted gene co-expression network analyses (WGCNAs) were performed using the WGCNA (v1.71) package in R (Langfelder and Horvath, 2008). This workflow is described in our GitHub (https://github.com/fmobegi/3-Tag-RNA-Seq-analysis). The DESeq2 *dds* normalized counts were transposed and then passed on to WGCNA to construct co-expression modules using the automatic network construction function ‘blockwiseModule()’ with default settings. The power was set at 10 after determining the optimum threshold using the ‘pickSoftThreshold()’ function (Supplementary Fig. S1). TOMType was signed. The correlation coefficient for module Eigengenes was calculated using Pearson’s correlation and hubs were selected using the ‘chooseTopHubInEachModule()’ function. The sorghum genome annotation file (Sbicolor_454_v3.1.1.gene.gff3) from the Phytozome repository (https://phytozome-next.jgi.doe.gov/info/Sbicolor_v3_1_1) was used to annotate WGCNA modules. Resulting gene IDs for each module were then subjected to ShinyGO V. 0.76.3 (Ge *et al.*, 2019) to obtain data on biological processes (BP) pathway enrichment using the classical method and Fisher’s exact test with a *P*-value threshold of *P*≤0.05 (Ge *et al.*, 2019). Pathway enrichments were visualized as dot plots generated using ggplot2 (Wickham, 2011) and pathway networks modules produced in Cytoscape_3.9.1 (Shannon *et al*., 2003). Furthermore, specific gene expression displayed as heatmaps was mapped on plant–pathogen interaction and phenylpropanoid pathways of the Kyoto Encyclopaedia of Genes and Genomes (KEGG) maps reconstructed using Lucidchart (https://www.lucidchart.com) and ChemDoddle (https://www.chemdoodle.com) for plant–pathogen interaction and phenylpropanoid pathways, respectively.

## Results

### Diverse phenotypes of *Striga* resistance in sorghum

**Fig. 1.**
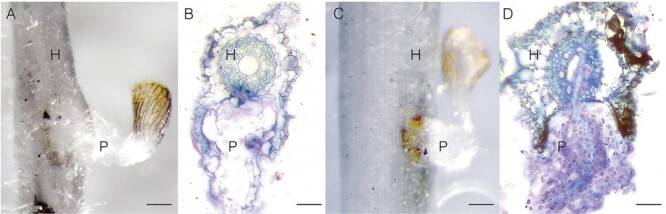
Contrasting phenotypes of *Striga* resistance. (A and B) Cell wall enhancement-based resistance in IS2814 showing parasite ingression blockage by cell wall barriers. (A) Close-up showing a poorly developed parasite. (B) A transverse section shows parasite tissue cycling around the host endodermis. (C and D) Hypersensitive reaction-based resistance in IS41724. (C) Parasite tissue starts a hypersensitive response at the interphase of the host and parasite. (C) Close-up shows a poorly developed parasite tissue and the beginning of necrosis. (D) Transverse sections show necrosis in areas of attempted penetration by the parasite. Scale bars=0.2 mm. The 5 μm thick sections were stained using 0.1% toluidine blue O dye. H=host, P=parasite.

We selected sorghum genotypes previously described in the study by Kavuluko *et al*. (2021). In that paper, we described N13 and IS9830 as *Striga* resistant based on the inability of the parasite to penetrate the host and establish vascular connections. This resistance was attributed to fortification of the host cell wall by various polysaccharides. In the same study, IS14963 was characterized as resistant due to elicitation of an intense HR leading to programmed cell death at the interface of the host and the parasite. Along with those genotypes, we also evaluate IS2814 and IS41724 whose mechanism of resistance had not been characterized previously. Our analysis showed that IS2814 had a resistance phenotype that blocked parasite ingression into the host endodermis. The parasite’s infectious organ—the haustorium—did not contact the host vasculature in the resistant genotype IS2814. Instead, the parasite haustorium bypassed the host endodermis, and emerged from host root tissue at the distal end (Fig. 1A). In IS41724, our analysis showed that programmed cell death occurred at the interfacee of the host and the parasite. A close-up of the parasite attaching to the host shows necrosis of a poorly developed parasitized seedling tissue at the interface of the parasite and the host. A transverse section through the haustorium confirmed the HR as the main resistance mechanism in this genotype (Fig. 1B).

These results show that *Striga* resistance in IS2814 and IS41724 is due to cell wall enhancement (CWE) and elicitation of a HR, respectively. In the end, the resistance phenotypes of sorghum used in the study were described as displaying either mechanical barrier resistance (N13, IS9830, and IS2814) or a HR (IS14963 and IS41724).

### Global transcriptome reprogramming in sorghum upon *Striga* infection

**Fig. 2.**
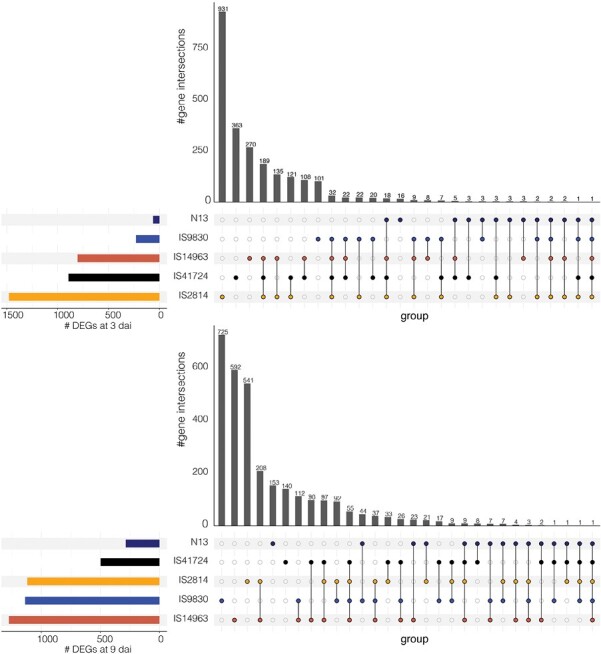
Transcriptional changes in sorghum upon *Striga* infection. UpSet plots showing how different genotypes of sorghum respond to infection by *S. hermonthica* at 3 dai (upper panel) and 9 dai (lower panel). The plot compares the overlap of differentially expressed genes (DEGs) between comparisons, with the colored horizontal bar graphs indicating the number of DEGs for each comparison. The black vertical bar graphs show the intersection size of DEGs, and the colored dots represent contributing comparisons from the differential expression analysis.

To understand the global transcriptome reprogramming that occurs following infection by *Striga*, and the underlying genetic causes of resistance for each genotype, we analyzed DEGs in each sorghum genotype at early (3 dai) and late stages (9 dai). We found unique genes that were differentially expressed for each genotype, suggesting a genotype-dependent transcriptional activation of defense genes. We also found DEGs that were shared among various genotypes and even core genes that were shared by all genotypes at each stage, suggesting that some transcriptional responses were common and conserved in sorghum (Fig. 2).

### WGCNA clarifies phenotypes of *Striga* resistance in sorghum

**Fig. 3.**
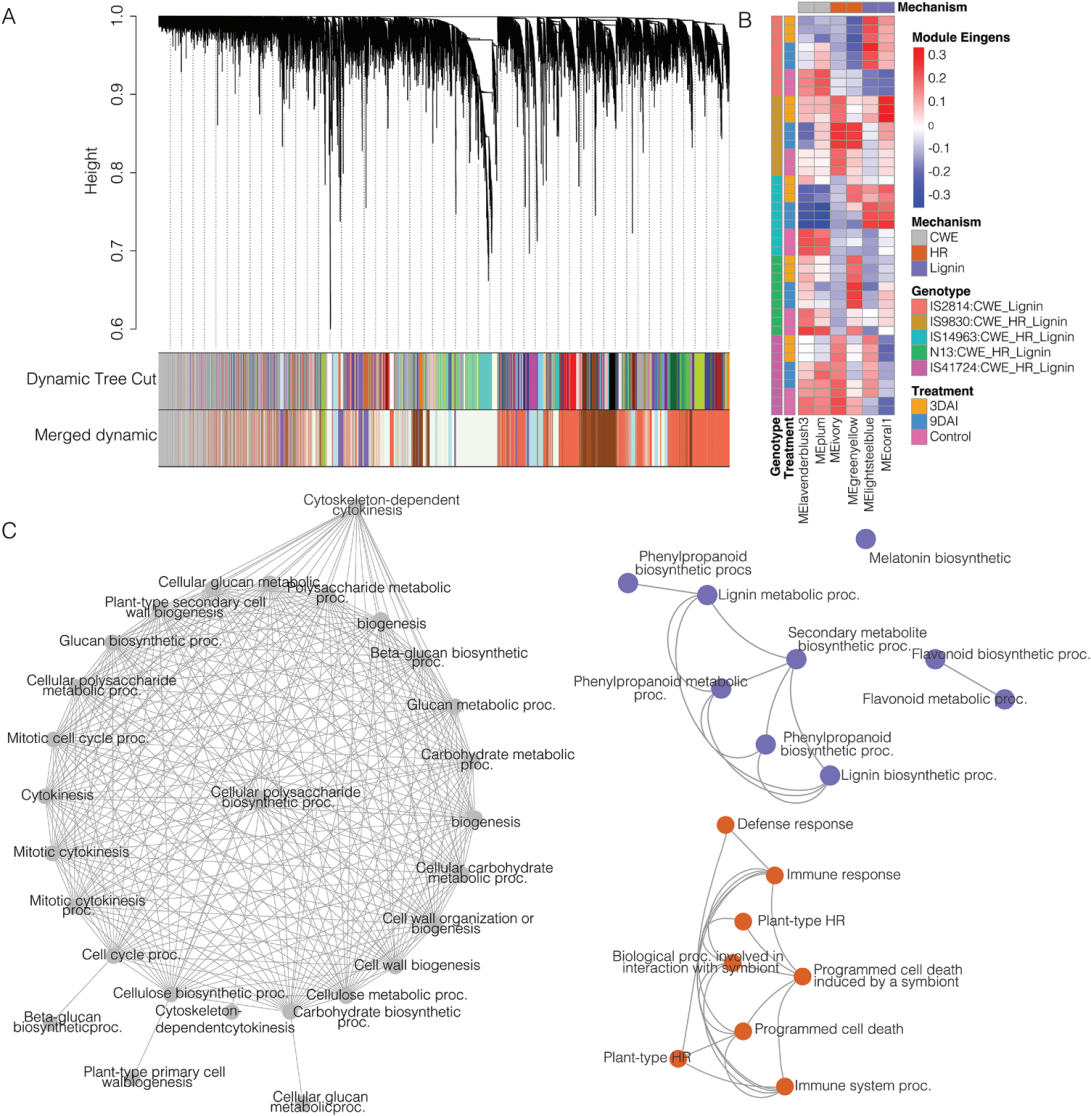
Weighted gene expression network analysis (WGCNA) of five *Striga*-resistant sorghum transcriptomes. (A) A dynamic cut tree of 70 and seven merged modules. (B) A heatmap showing clarification of mechanisms of resistance using WGCNA module Eigengene values. Six modules corresponded to three mechanisms of *Striga* resistance. Cell wall enhancement (CWE), hypersensitive response (HR), and secondary metabolites including lignin biosynthesis. The heatmap also shows that WGCNA also partitioned genotypes according to modes of resistance. (C) Pathway interaction networks representing *Striga* resistance mechanisms: MElavendablush3 representing significant enrichment for primary and secondary cell wall reinforcements, most notably callose apposition though β-glucan biosynthesis. MEgreen-yellow module showing network processes for programmed cell death, and MEcoral1 showing enrichment for secondary metabolite biosynthesis including lignin and melatonin. Network nodes are color-coded according to the modes of resistance.

To further resolve the complexity of resistance responses in the various sorghum phenotypes, we performed a WGCNA. This analysis clusters DEGs based on their expression levels in different samples, and groups them as modules. Thus, WGCNA allowed us not only to identify modules associated with various modes of resistance in individual genotypes, but to also to group sorghum genotypes showing similar gene expression patterns and identify critical genes for each regulatory network (Fig. 3).

We found a total of 79 modules of which 77 were statistically significant at *P*≤0.05 (Supplementary Dataset S1). A Gene Ontology (GO) enrichment analysis of annotated modules (Supplementary Dataset S2) revealed that six modules were enriched for processes of: (i) CWE; (ii) HR; and (iii) production of secondary metabolites including lignin biosynthesis (Fig. 3; Supplementary Fig. S2). CWE pathways were enriched in the MElavenderblush3 and MEplum modules for processes of cell wall growth, such as cell wall biogenesis, cell wall organization, and microtubule development. Additionally, the modules were enriched for the synthesis of cell wall polysaccharides, for example β-glucan and cellulose biosynthesis. HR mechanism pathways were in the MEivory and MEgreen-yellow modules. Here, processes of programmed cell death, HR, and immune activation consistent with host–pathogen interactions were enriched in both modules, but more notably in MEivory. Finally, MElightsteel blue and MEcoral1 were enriched for pathways consistent with the production of secondary metabolites that enhance resistance against invading pathogens. In these modules, we found enrichment for upstream metabolites of lignin biosynthesis—the phenylpropanoid pathway as well as flavonoids. Notably, MEcoral1 was also enriched for melatonin biosynthesis. This finding was unexpected, but significant because, as it happens, melatonin increases lignification (Zhao *et al*., 2022).

We grouped genotypes showing similar molecular signatures of *Striga* resistance and found consistent clustering of genotypes and treatments according to module Eigengene values. This allowed us to assign resistance mechanisms for the various genotypes as follows: (i) IS2814 was strongly positively correlated with modules for cell wall enhancement and lignification; and (ii) IS9830, IS14963, N13, and IS41724 were all correlated with the three modes of resistance (CWE, HR, and lignification) albeit to different degrees (Fig. 3).

The final aspect of our WGCNA was to identify transcriptional regulatory pathways and associated top network genes in the various individual and merged modules involved in *Striga* resistance. A list of annotated top genes in each module is provided in Supplementary Table S1. We would like to highlight three of these.

The first of these is Sobic.001G529600, in the green module. This gene encodes glucan synthase-like 10 (GSL10), a member of the glucan synthase-likes (GSLs). GSLs are known to catalyze the synthesis of the cell wall component callose, which provides structural cell wall reinforcement against pathogen infection (Bacete *et al*., 2018). A closer look at the enriched pathways in the green module revealed significant enrichment for pathways involving innate immune response activating receptor signaling, immune response receptor transduction, and activation of immune responses—suggestive of a PAMP-induced callose deposition (Supplementar Fig. S2). Consistent with callose deposition, the green-yellow network was enriched for β-glucan, xylan, and other carbohydrates that converged at the polysaccharides.

The second gene is Sobic.004G321100 in the green-yellow module. This gene encodes a phosphoinositide phosphatase and forms an important component in the phosphoinositide signaling-mediated defense network for both basal and systemic responses (Hung *et al*., 2014). Consistent with this view, we found significant enrichment of glycolipid and phospholipid biosynthetic and metabolic processes. Reinforcing our hypothesis, we found significant enrichment of cell death processes in the module (Supplementary Fig. S2).

The third gene is Sobic.006G280000 in the turquoise module which encodes the pathogenesis-related thaumatin-like family of genes, consistent with activation of the basal immune response to pathogens. It is known that plants produce pathogenesis-related (PR) proteins that play key roles in plant disease resistance responses, specialized in systemic acquired resistance (SAR) (Hamamouch *et al*., 2011). As expected, pathways for responses to wounding and basal-triggered immunity were enriched in this module (Supplementary Fig. S2).

### *Striga* activates multiple defense pathways in sorghum

To determine specific defense responses that *Striga* triggers in sorghum, we studied gene expression patterns of sorghum in the key pathogen detection, signaling, and defense pathways that included (i) plant–pathogen interaction; (ii) phenylpropanoid and (iii) hormone signaling pathways based on KEGG maps.

#### *Striga* elicits both pathogen- and effector-triggered immunity in sorghum

**Fig. 4.**
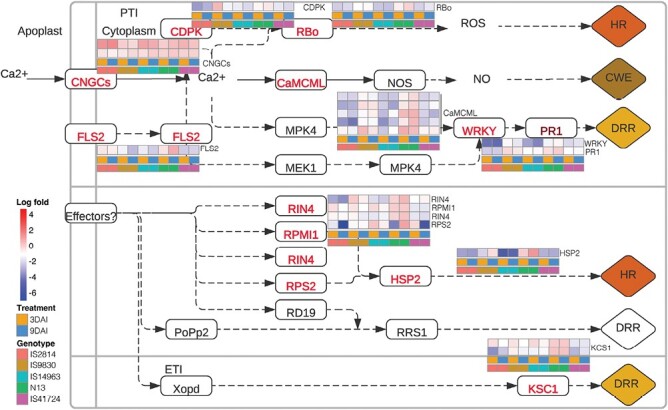
*Striga* elicits both pathogen- and effector-triggered immunity in sorghum. Gene expression patterns of sorghum genotypes in the plant–pathogen interaction Kyoto Encyclopedia of Genes and Genomes (KEGG) map. *Striga* induced pathogen- and effector-triggered immunity and showed differential gene expression patterns in the sorghum genotypes for various pathogen immune responses to various degrees. In IS9830 and N13, genes were induced for hypersensitive response (HR) as well as systemic acquired resistance (SAR) though the WRKY signaling pathway. The red-colored font of enzymes indicates the DEGs in response to *S. hermonthica* infestation. A red box denotes up-regulated expression while a blue box denotes down-regulated expression.

In this pathway, both PTI and ETI are activated, leading to downstream defense processes (Fig. 4). On PTI, our results showed that in all genotypes CNGCs, involved in Ca^2+^ sensing, were induced at all stages. However, CDPK and RBo genes leading to HRs were only induced in IS9830 and N13. CaM/CML and FLS2 that lead to CWE and other disease response reactions including WRKY and PR1-dependent disease responses were induced only in IS9830. On ETI, resistance genes RIN4, RPMI, RPS2, and HSP2 were induced in all genotypes—but more notably in N13 and IS9830. RPS2 was also notably induced in IS41724 at 3 dai. In all cases, ETI genes were not induced in IS2814. These expression patterns suggest that ETI responses are induced in N13, IS9830, IS14963, and IS41724, and that PTI is induced in all genotypes for upstream genes but more notably in N13 and IS9830 for downstream genes. This observation is consistent with module enrichment results that grouped N13 and IS9830 as displaying both cell wall-based resistance and HRs. These results are also consistent with phenotypic observations that showed HR responses in IS41724.

#### Lignin biosynthesis pathway is critical against *Striga* resistance in sorghum

**Fig. 5.**
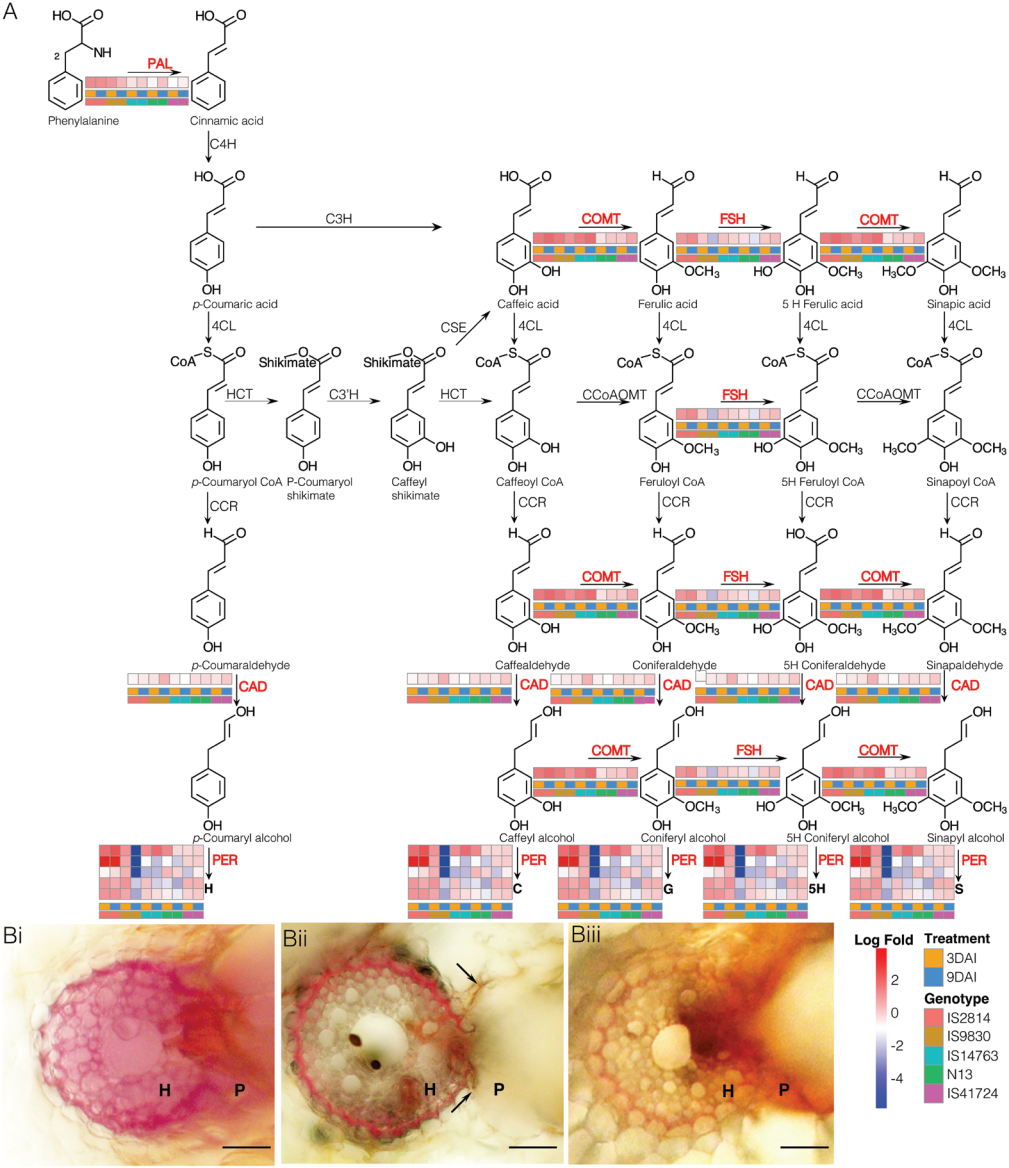
Lignification as a defense mechanism against *Striga* in sorghum. (A) Differential gene expression in the phenylpropanoid biosynthetic pathway leading to H, C, G, 5H, and S types of lignin. Key lignin genes were positively regulated in IS2814 and N13 compared with other genotypes. The red-colored font of enzymes indicates the DEGs in response to *S. hermonthica* infestation. A red box denotes up-regulated expression while a blue box denotes down-regulated expression. (B) Histochemical staining using phloroglucinol-HCl for lignin detection. Varying red color intensity in vibratome (20 μm thick) sections in: (Bi) N13, deepest coloration indicating high lignification, (Bii) IS41724 moderate staining also showing necrosis at the host–parasite interface (arrows) characteristic of a hypersensitive response (HR). and (Biii) IS14963 showing faint lignin staining but intense staining at the host–parasite interface (arrow). Scale bar is 0.1 mm; H=host; P=parasite. Pathway enzymes: PAL, l-phenylalanine ammonia-lyase; C4H, cinnamate 4-hydroxylase; C3H, 4-coumarate 3-hydroxylase; COMT, caffeate/5-hydroxyferulate 3-*O*-methyltransferase; F5H, ferulate 5-hydroxylase/coniferaldehyde 5-hydroxylase; 4CL, 4-hydroxycinnamate:CoA ligase, HCT, 4-hydroxycinnamoyl CoA:shikimate/quinate hydroxycinnamoyltransferase; C3ʹH 4-coumaroyl shikimate/quinate 3ʹ-hydroxylase; CSE, caffeoyl shikimate esterase; CCoAOMT, caffeoyl CoA 3-*O*-methyltransferase; CCR, cinnamoyl CoA reductase; CAD, cinnamyl alcohol dehydrogenase; and PER, peroxidase. The pathway map was constructed using data from Barros *et al.* (2019).

Next, we looked at the phenylpropanoid pathway that leads to lignin biosynthesis (Fig. 5A). Modules enriched for lignin biosynthesis (lightbluesteel and coral1) had the following annotated genes: one phenylalanine ammonia lyase (PAL), ferulate 5-hydroxylase (F5H), caffeic acid *O*-methyl transferase (COMT), cinnamyl alcohol dehydrogenase (CAD), and five peroxidase genes. All these genes were significantly induced in IS2814, N13, and IS9830 in early and late stages of infection. The induction was more notable in IS2814 compared with N13 and IS9830. In comparison, induction levels of the genes remained lower in IS41724 and IS14963. These findings reinforced WGCNA results that showed significant enrichment of lignin biosynthesis in IS2814, N13, and IS9830. To further examine lignin as a defense mechanism, we performed lignin staining assays on N13, IS41724, and IS14963. Consistent with gene expression results, lignin staining was highest in N13, moderate in IS41724, and almost undetectable in IS14963 (Fig. 5B).

#### *Striga* activates jasmonic acid and salicylic acid immune responses in sorghum

**Fig. 6.**
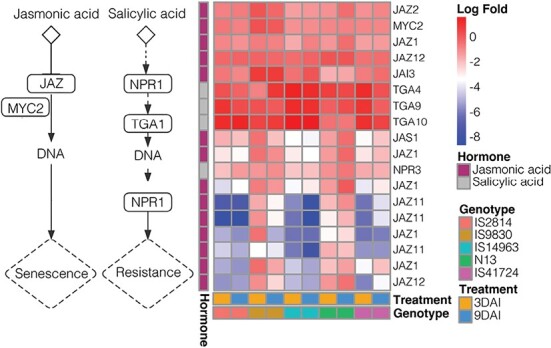
*Striga* activates both jasmonic acid (JA) and salicylic acid (SA) downstream immune responses in sorghum. Differential activation of JA and SA genes in different *Striga* genotypes. IS9830 and N13 showed a marked induction of JA responses. In contrast, SA responses were induced in all genotypes.

Lastly, we examined the hormone signaling pathway focusing on jasmonic acid (JA) and salicylic acid (SA) signaling (Fig. 6). JA triggers the degradation of JASMONATE ZIM DOMAIN (JAZ) transcriptional repressor proteins, leading to release of downstream transcription factors that in turn activate defense responses including programmed cell death. Correspondingly, JA-mediated defense is followed by suppression of JAZ genes. Consistent with JA activation, JAZ expression levels were 4- to 8-fold lower in nine JAZ proteins for the genotypes IS2814, IS14963, and IS41724. In the other two genotypes, IS9830 and N13, JAZ levels remained the same. The levels of MYC2 were also unchanged. Interestingly, levels of SA-associated hormones remained high in all genotypes. These results indicate that JA mediates genotype-specific *Striga* resistance responses in genotypes IS2814; IS14963 and IS41724 exhibit *Striga* resistance in a JA-dependent manner; and *Striga* triggers an SAR in all the genotypes.

## Discussion

We sought to clarify genetic causes of different modes of *Striga* resistance in sorghum. We used fiv sorghum genotypes with distinct documented mechanisms or resistance. Three of the genotypes had mechanical resistance and two had HRs. We found that sorghum displayed many DEGs for each of the genotypes (Fig. 2), underscoring the fact that sorghum has a large genetic pool that can be exploited for improvement. Bearing in mind that each genotype had a different biological status: IS41724 and N13 are Dura, IS9830 and IS14963 are Caudatum, and IS2814 is a Durra-Caudatum sorghum (Kavuluko *et al*., 2021), and that the genotypes were domesticated in different regions of Africa (Kavuluko *et al*., 2021), unique sets of genes responding to infection by the parasite suggest independent adaptation and evolution of the resistance, and shared genes shows conservation of key pathways for resistance against the parasite.

### Do sorghum genotypes harbor distinct resistance mechanisms against *Striga*?

We used WGCNA to further decipher these complexities. In this analysis, reads are weighted based on similarity of gene expression and assigned modules based on this similarity. We found that WGCNA efficiently delineated mechanisms of *Striga* resistance and placed them in modules bearing common molecular signatures of resistance (Fig. 3). From these groupings, we were able to classify the genotypes according to resistance mechanisms based on activated pathways. WGCNA grouping bore truth to earlier observations and provided new insights into sorghum resistance against *Striga*. For example, N13 has been described as having quantitative resistance based on cell wall enhancement traits (Maiti *et al*., 1984; Haussmann *et al*., 2004); in this study, IS2814 was shown to harbor mechanical barrier resistance, and IS41724 and IS14963 were shown to display HR resistance.

### Can WGCNA help pinpoint key *Striga* resistance genes in sorghum?

An additional feature of our WGCNA was identification of top genes regulating module networks. We focused on three key genes based on their documented role in plant–pathogen interactions.

The first gene of interest GLS10 is a key regulator of callose biosynthesis. Callose is a β-(1,3)- d-glucan widely distributed in higher plants. In addition to its role in normal growth and development, callose plays an important role in plant defense (Voigt, 2014). In Arabidopsis, deposition of callose or papillae at sites of fungal penetration serves as an early response of host plants to microbial attack providing protection against entry of the fungus (Jacobs *et al*., 2003). Because the green module network was also enriched with immune responses, we attributed this to a PAMP-induced GLS callose deposition as earlier described (Jacobs *et al*., 2003). This defense response is characterized by formation of callose-rich cell wall appositions called papillae (Jones and Dangl, 2006; Schwessinger and Ronald, 2012). Because of the earlier documented role of papillae in stopping penetration of fungal hyphae in barley (Chowdhury *et al*., 2014), it is not far-fetched to speculate that callose apposition may make the difference between a successful and a failed *Striga* penetration attempt. Indeed, overexpression of GSL5/PMR4 leads to enhanced papillary callose deposition and complete penetration resistance to powdery mildew, showing that cell wall carbohydrates can contribute to penetration resistance (Ellinger *et al*., 2013).

The second interesting gene, Sobic.004G321100, in the green-yellow module encodes a phosphoinositide phosphatase and forms an important component in the phosphoinositide signaling-mediated defense network for both basal and systemic responses (Hung *et al*., 2014). Therefore, identification of this gene was indicative of a phosphoinositide signaling for basal and systemic response following *Striga* infection. The membrane-associated phospholipids along with the soluble inositol phosphates (collectively known as phosphoinositides) are present in all eukaryotic cells and are implicated in plant responses to many environmental stimuli (reviewed in Stevenson *et al*. (2000). The phosphoinositide pathway and inositol-1,4,5-triphosphate (InsP3) have been implicated in mediating both basal defense and SAR responses (Hung *et al*., 2014). The study showed that flagellin-induced Ca^2+^ release as well as the expression of some flg22-responsive genes were attenuated in the InsP 5-phosphatase plants, which had reduced InsP3, making them more susceptible. Additionally, InsP 5-phosphatase plants were more susceptible to virulent and avirulent strains of *Pseudomonas syringae* pv. *tomato* (*Pst*) DC3000 and had lower basal SA levels, and the induction of SAR in systemic leaves was reduced and delayed. Plausibly, phosphoinositide signaling is one component of the plant defense network and is involved in both basal and systemic responses. Confirmation of this process can also be drawn from the significant enrichment of cell death processes in this module.

The third gene, Sobic.006G280000, in the turquoise module, encodes the pathogenesis-related thaumatin-like family of genes. Production of PR proteins represents the hallmark of SAR in plants (Hamamouch *et al*., 2011). Specifically, thaumamatin-like proteins (TLPs) can rapidly accumulate to high levels in response to biotic or abiotic stress and exhibit antifungal activity in various plant species (Petre *et al*., 2011). As expected, overexpression of TLPs can induce resistance against pathogens. For example, overexpression of TLP has been shown to increase resistance against fungal pathogens in tomatoes (Liu *et al*., 2012) and potato (Acharya *et al*., 2013). Based on the gene expression patterns observed in our studies, we infer that TLPs would be good candidates to impart *Striga* resistance. Support for this hypothesis can be drawn from gene expression studies that have shown up-regulation of PR genes in the resistant rice variety (Nipponbare) relative to the susceptible IAC (Swarbrick *et al*., 2008). In the report of Swarbrick *et al*. (2008), genes encoding endochitinases (PR-3), glucanases (PR-2), and TLPs (PR-5) were up regulated in the resistant rice variety. Furthermore, a subsequent similar study in cowpea–*Striga* interactions showed that levels of PR5 were dramatically up-regulated in the resistant variety (Huang *et al*., 2012).

### *Striga* elicits both pathogen- and effector-triggered immunity in sorghum

Individual gene expression further supported activation of PTI and ETI for various modes of resistance (Fig. 4). At the PTI level, there was up-regulation of HR genes, CNGC, CDPK, and RBO, and SAR-associated genes, WRKY and PR1 in IS9830 and N13 consistent with the modes of resistance of these genotypes. At the ETI level, resistance genes RIN4, RPMI, RPS2, as well as HSP and KSC were up-regulated in IS9830 and N13. Downstream pathways of ETI and PTI led to activation of defense processes such as CWE that were also consistent with the modes of resistance for the genotypes. The concept of an active PTI triggered by *Striga* infection raised the question as to what sorghum recognizes as a PAMP. We hypothesized that products of cell wall degradation created upon *Striga* infection act as elicitors of PTI akin to damage-associated pathogen patterns (DAMPs) previously described (Souza *et al*., 2017), but also plausibly *Striga* has PAMPs. The latter hypothesis is reinforced by identification of a cell wall-localized glycine-rich protein that acts as a PAMP in the parasitic plant *Cuscuta reflexa* (Slaby *et al*., 2021).

### Lignin biosynthesis pathway is critical against *Striga* resistance in sorghum

We examined the phenylpropanoid pathway that leads to lignin biosynthesis and found up-regulation of key lignin biosynthesis genes—PAL, COMT, FSH, CAD, and peroxidases—in IS2814, IS9830, and N13 that exhibited cell wall-based resistance modes (Fig. 5). In concert, an increased expression of lignin genes has been reported in cowpea infected with *S. gesnerioides* (Huang *et al*., 2012) and in *S. hermonthica*–rice interactions (Swarbrick *et al*., 2008; Mutuku *et al.*, 2019), suggesting that lignification is a critical mechanism of defense employed by hosts to stop *Striga* parasitism.

In the defense signaling pathway, the most differentially induced genes were those involved in JA signaling and response, particularly JAZ—and this expression pattern corresponded with the resistance modes (Fig. 6). Notably, in IS9830 and N13, JAZ genes were notably induced. In striking contrast, SA response genes, NP3 and TGA, were induced in all genotypes, suggesting active JA and SA signaling in the sorghum genotypes. In a previous study (Hiraoka and Sugimoto, 2008) of differential transcriptional response of susceptible and moderately resistant sorghum to *S. hermonthica*, the authors found that *Striga* parasitism induced JA-responsive genes and suppressed SA-responsive genes in the roots of a highly susceptible cultivar and suggested that susceptible hosts recognize *Striga* parasitism as wounding stress rather than microbial stress. In contrast, they found that in the roots of a moderately resistant sorghum cultivar, both SA- and JA-responsive genes were induced, suggesting that resistance involved pathways associated with both wounding and pathogen challenge. In *Striga*–rice interaction, Mutuku *et al.* (2015) reported that both JA and SA pathways were induced, but the induction of the JA pathway preceded that of the SA pathway, and that foliar application of JA resulted in higher resistance. These seemingly contrasting findings suggest that JA and SA signaling pathways are host–pathogen specific, but such a hypothesis will require further investigation.

## Conclusions

Taken together, our analysis of sorghum resistance to *S. hermonthica* is suggestive of diverse mechanisms for surveillance, signaling, and defense. We identified important candidate genes that impinge on these processes that eventually impart *Striga* resistance. This makes future sorghum improvement for *Striga* resistance feasible and possible through breeding or genetic modification approaches.

## Supplementary data

The following supplementary data are available at *JXB* online.

Table S1. Top network genes for single and merged modules.

Fig. S1. Network topology for soft threshold power.

Fig. S2. Enrichment analysis for key network genes.

Dataset S1. A spreadsheet of module Eigengene values.

Dataset S2. A spreadsheet of module classifications, gene IDs, and annotations.

erad210_suppl_supplementary_table_S1_figures_S1-S2

erad210_suppl_supplementary_Dataset_S1

erad210_suppl_supplementary_Dataset_S2

## Data Availability

Raw reads from transcriptome sequencing have been deposited in the National Center for Biotechnology Information (NCBI) Sequence Read Archive under BioProject accession number PRJNA906112.

## Abbreviations

CWE: cell wall enhancement
DEG: differentially expressed gene
ETI: effector-triggered immunity
HR: hypersensitive response
PTI: pathogen-triggered immunity
SAR: systemic acquired resistance
SSA: sub-Saharan Africa
TLP: thaumamatin like protein
WGCNA: weighted gene co-expression network analysis
