# Supplementary material for: Resolving intergenotypic Striga resistance in sorghum
Source: J Exp Bot. 2023 Jun 1;74(17):5294–306. doi: 10.1093/jxb/erad210 (PMC10498017; doi:10.1093/jxb/erad210)
Supplement: erad210_suppl_supplementary_table_S1_figures_S1-S2 [file erad210_suppl_supplementary_table_s1_figures_s1-s2.pdf]

Supplementary data

Resolving intergenotypic *Striga* resistance in sorghum

Running title: *Striga* resistance in sorghum

Sylvia Mutinda[1, 2], Fredrick M. Mobegi[3], Brett Hale[4], Olivier Dayou[5], Elijah Ateka[6], Asela Wijeratne[4], Susann Wicke[5], Emily S. Bellis[7*], and Steven Runo[2, 5*]

[1]Pan African University Institute for Basic Sciences, Technology and Innovation, Nairobi, Kenya
[2]Department of Biochemistry, Microbiology and Biotechnology, Kenyatta University, Nairobi, Kenya
[3]Department of Clinical Immunology, PathWest Laboratory Medicine WA, Fiona Stanley Hospital Network, Murdoch, Western Australia
[4]Department of Biological Sciences, Arkansas State University, Jonesboro, Arkansas, USA
[5]Institute for Biology, Humboldt University, Germany
[6]Department of Horticulture, Jomo Kenyatta University of Agriculture and Technology, Nairobi, Kenya
[7]Department of Computer Science, Arkansas State University, Jonesboro, Arkansas, USA

Author email: Sylvia Mutinda (sylvia.mutindah@gmail.com); Fredrick M. Maati (frederick.mobegi@health.wa.gov.au), Brett Hale (brett.hale@smail.astate.edu), Olivier Dayou (olivierdayou2@gmail.com), Elijah Ateka (emateka@yahoo.com), Asela Wijeratn (awijeratne@astate.edu), Susann Wicke (susann.wicke@hu-berlin.de)

[*]Corresponding author email: runo.steve@ku.ac.ke and ebellis@astate.edu)

ORCID: Sylvia Mutinda (https://orcid.org/0000-0001-6887-647X); Fredrick M. Maati (https://orcid.org/0000-0003-0554-9919), Brett Hale (https://orcid.org/0000-0002-4984-017X), Olivier Dayou (https://orcid.org/0000-0002-3716-3714), Asela Wijeratn (https://orcid.org/0000-0002-7817-6351v), Susann Wicke (https://orcid.org/0000-0001-5785-9500), Emily S. Bellis (https://orcid.org/0000-0001-6066-1466), Steven Runo (https://orcid.org/0000-0002-9043-2338)

Supplementary table 1: Top-network genes for single and merged modules

Gene ID	Module	Arabidopsis ortholog Annotation
Sobic.002G198200	blue	TBP-associated factor 2
Sobic.001G277900	brown	N/A
Sobic.005G104500	brown	phosphatidylinositol 4-OH kinase beta1
Sobic.003G199300	darkgreen	ARM repeat superfamily protein
Sobic.001G474600	darkgreen	Calmodulin-binding transcription activator protein with CG-1 and Ankyrin domains
Sobic.001G161900	darkgreen	DREB2A-interacting protein 2
Sobic.004G106400	darkgreen	Mitogen activated protein kinase kinase kinase-related
Sobic.010G094600	darkgreen	RING/U-box superfamily protein
Sobic.001G290000	darkgreen	beta-hydroxyisobutyryl-CoA hydrolase 1
Sobic.010G261500	darkolivegreen	calcineurin B subunit-related
Sobic.004G304800	darkolivegreen	membrane-associated progesterone binding protein 3
Sobic.002G404700	darkorange	chromatin remodeling 5
Sobic.009G053900	darkorange	ubiquitin protein ligase 6
Sobic.003G244200	darkorange2	vacuolar ATP synthase subunit E1
Sobic.009G083600	darkseagreen4	EF hand calcium-binding protein family
Sobic.004G104900	floralwhite	Protein kinase superfamily protein
Sobic.001G529600	green	glucan synthase-like 10
Sobic.004G321100	green-yellow	Phosphoinositide phosphatase family protein
Sobic.003G103200	lightgreen	peroxin 14
Sobic.004G313300	lightgreen	N/A
Sobic.004G093600	purple	Dihydrolipoamide acetyltransferase, long form protein
Sobic.002G040700	purple	Tetratricopeptide repeat (TPR)-like superfamily protein
Sobic.010G251200	purple	UDP-sugar pyrophosphorylase
Sobic.004G160700	purple	TUDOR-SN protein 1
Sobic.004G146500	purple	CTC-interacting domain 4
Sobic.002G244100	purple	Protein of unknown function (DUF1664)
Sobic.004G307500	purple	phenylalanyl-tRNA synthetase, putative / phenylalanine--tRNA ligase, putative
Sobic.006G280000	purple	transportin 1
Sobic.006G217300	skyblue3	NAD(P)-binding Rossmann-fold superfamily protein
Sobic.001G455700		microtubule-associated proteins 70-2
Sobic.001G106600	steelblue	Sulfite exporter TauE/SafE family protein
Sobic.007G051700	steelblue	Transducin family protein / WD-40 repeat family protein
Sobic.004G013300	steelblue	glutamate receptor 3.3
Sobic.001G534800	tan	Adaptor protein complex AP-2, alpha subunit
Sobic.004G127700	tan	1,2-alpha-L-fucosidases
Sobic.010G278300	tan	Prolyl oligopeptidase family protein
Sobic.005G003300	tan	Clathrin, heavy chain
Sobic.002G355800	tan	Mo25 family protein
Sobic.001G122832	tan	MAPK/ERK kinase kinase 1
Sobic.006G127600	tan	SIGNAL PEPTIDE PEPTIDASE-LIKE 3
Sobic.007G226300	turquoise	Myosin heavy chain-related protein
Sobic.001G034100	turquoise	poltergeist like 4
Sobic.001G267600	turquoise	Pathogenesis-related thaumatin superfamily protein
Sobic.006G032200	white	Heavy metal transport/detoxification superfamily protein
Sobic.009G082600	white	proline extensin-like receptor kinase 1

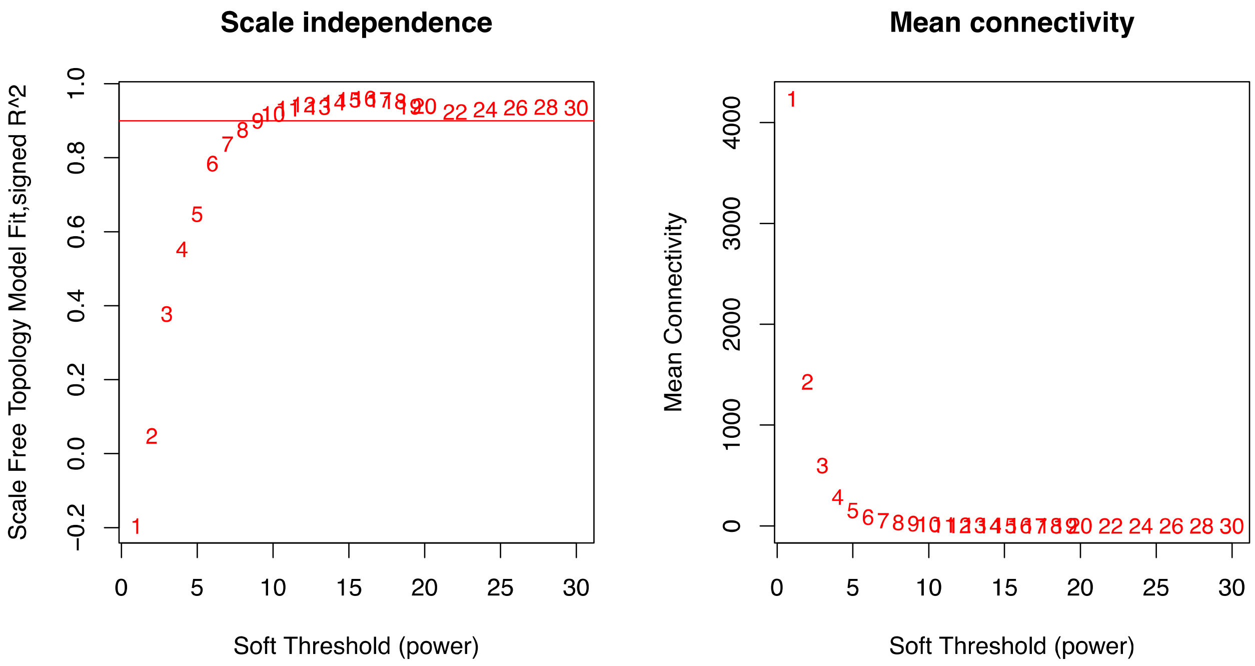

Supplementary Figure 1. Network topology for soft threshold power. Plots of both scale-free fit index as a function of soft threshold power and mean connectivity as a function of soft threshold power are shown.

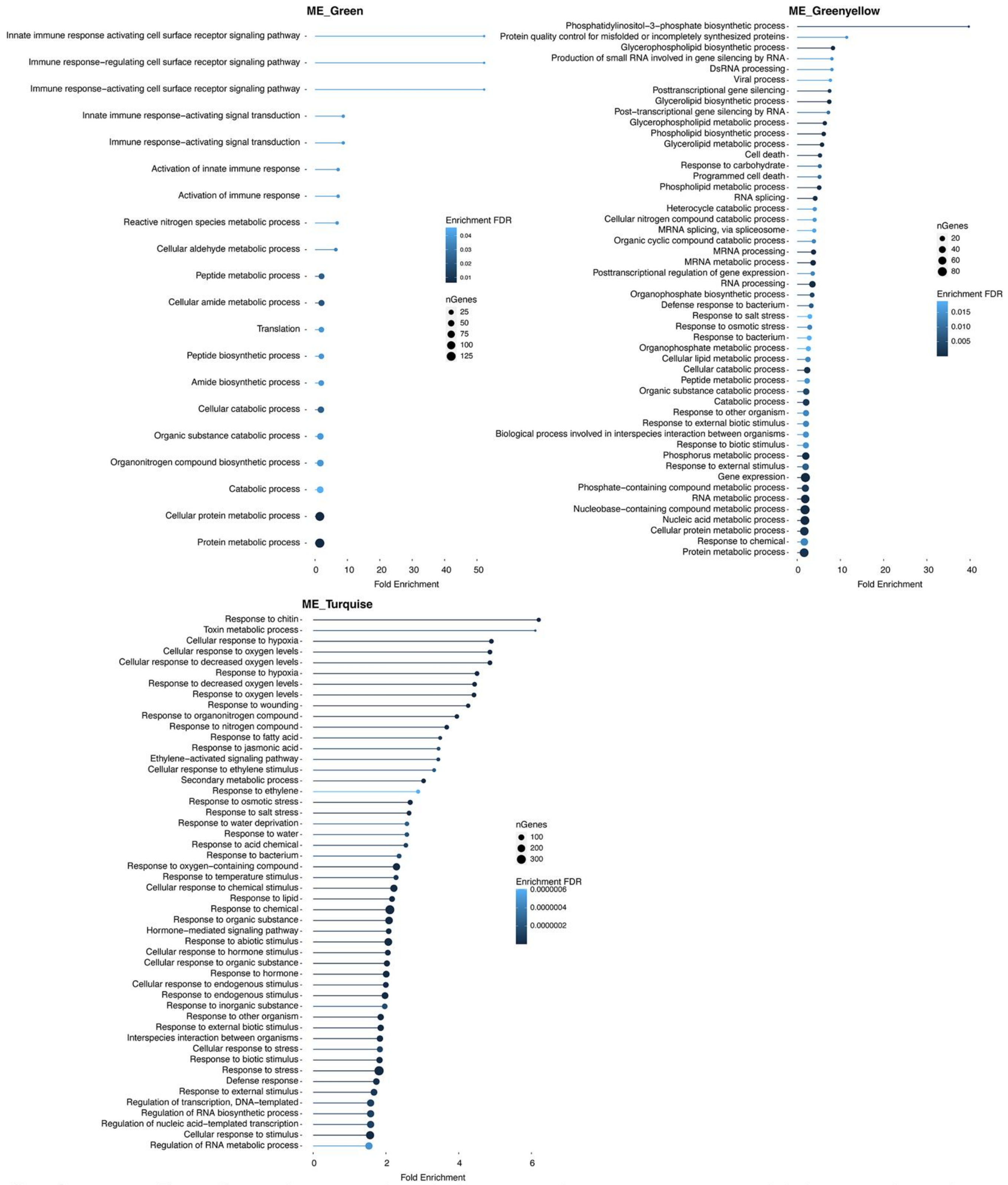

Supplementary Figure 2. Enrichment analysis for key network genes. (A) Green module key gene beta glucan showing immune response pathways, (B) green-yellow module with phosphoinositide signaling and enrichment for programmed cell death processes, and (C) turquoise module showing enrichment for pathogen defense responses.
